# Risk assessment as a tool for improving external biosecurity at farm level

**DOI:** 10.1186/s12917-015-0477-7

**Published:** 2015-07-28

**Authors:** Susanna Sternberg Lewerin, Julia Österberg, Stefan Alenius, Marianne Elvander, Claes Fellström, Madeleine Tråvén, Per Wallgren, Karin Persson Waller, Magdalena Jacobson

**Affiliations:** Department of Biomedical Sciences and Veterinary Public Health, Swedish University of Agricultural Sciences, Box 7036, 75007 Uppsala, Sweden; National Veterinary Institute, 75189 Uppsala, Sweden; Department of Clinical Sciences, Swedish University of Agricultural Sciences, Box 7054, 75007 Uppsala, Sweden

**Keywords:** On-farm biosecurity, Risk assessment, Infectious disease, Livestock, Disease introduction, Model

## Abstract

**Background:**

Biosecurity routines at herd level may reduce the probability of introduction of disease into the herd, but some measures may be regarded as expensive and cumbersome for the farmers. Custom-made measures based on individual farm characteristics may aid in improving the actual application of on-farm biosecurity.

The aim of the study was to provide a tool for calculating the effects of different biosecurity measures and strategies on the individual farm level.

A simple model was developed to assess the risk of disease introduction and the need for biosecurity measures in individual farms. To illustrate the general applicability of the tool, it was applied to theoretical examples of Swedish cattle and pig farms and diseases endemic in those animal species in the EU, in two scenarios with different between-farm contact patterns.

**Results:**

The model illustrated that the most important factors affecting the risk, and the effect of biosecurity measures such as quarantine routines and protective clothing, were the frequency of between-farm contacts and prevalence of the disease. The risk of introduction as well as the effect of biosecurity measures differed between farm types and disease transmission routes. Adapting contact patterns to mitigate a specific disease risk was as important as biosecurity measures for some farm types, but the largest effect was seen when combining biosecurity measures with more planned contact patterns.

**Conclusions:**

The risk assessment model proved useful for illustrating the risk of introduction of endemic diseases and the mitigating effect of different biosecurity measures on farm level. Model outputs could be used to justify prioritisation of measures or adapting contact patterns. The theoretic exercise of adjusting model inputs and comparing outputs may help veterinary advisors to understand farm-specific risks and motivate farmers to improve biosecurity in their individual farm, as it can be tailored to each farmer’s needs and preferences.

## Background

Endemic as well as exotic contagious diseases may cause devastating outbreaks in individual herds of livestock. Infectious agents may be transmitted between farms by various routes such as live animals, trucks and other vehicles, people, aerosols, fomites, wildlife and insect vectors [1]. The frequency and contact patterns of these transmission routes are important determinants for the risk of disease introduction and thus also important for the epidemiological investigation of a disease outbreak [2].

Biosecurity routines at herd level may reduce the probability of introduction of disease into the herd. For example, the prevalence of Aujezsky’s disease, porcine reproductive and respiratory syndrome, *Mycoplasma hyopneumoniae*, bovine coronavirus, and infectious bovine rhinotracheitis has been associated with the level of biosecurity at farm level [3–7]. Following the Swedish ban of antimicrobial feed additives in 1986, arising problems with infectious diseases in pig herds were mainly controlled by improving external and internal biosecurity in the production [8, 9]. Conversely, a high prevalence of infectious diseases has been used as a proxy for low biosecurity [10]. As most diseases have a negative impact on the well-being of the animals, their productivity and thereby also the economy of the farmer, there are incentives for stringent biosecurity routines. The new European Animal Health Regulation emphasises the responsibility of farmers for preventive measures, including on-farm biosecurity, in order to control contagious diseases within the European Union (EU) [11]. New Swedish legislation on the prevention of zoonoses also puts responsibility on farmers for providing biosecurity measures to prevent spread of zoonotic agents to and from people visiting their farm [12] and Danish legislation requires all farmers with large dairy holdings to set up an approved biosecurity plan for their herd [13]. Moreover, disease prevention in the form of improved on-farm biosecurity plays a part in many national and regional initiatives within the EU. These efforts to turn disease control towards on-farm prevention put extra demands on the advisory function of the veterinary profession. A risk assessment tool could be helpful in developing skills in risk-based prioritisation of external farm biosecurity measures among veterinary practitioners and achieving a trustful dialogue with farmers on biosecurity.

Farmers may regard biosecurity measures as expensive and cumbersome and the biosecurity routines on farm level are not always optimal [13–17]. Moreover, prioritisation of measures based on individual farm characteristics may aid in improving the actual application of on-farm biosecurity. Farmers may want to discuss different biosecurity measures separately, to justify investments in time and money, but estimates of the preventive effect of individual measures in different situations are difficult to obtain.

The aim of this study was to create a tool for on-farm risk based prioritisation of commonly recommended biosecurity measures, as an aid to veterinarians, animal health organisations and farmers in their strategies to improve on-farm biosecurity.

## Methods

A simple model was used to assess the risk of disease introduction, the need for and potential effect of biosecurity measures in individual farms. To illustrate the general applicability of the tool, it was applied to theoretical examples of Swedish cattle and pig farms and diseases endemic in those animal species in the EU. The tool was created as a stochastic model. A deterministic version of the model was also built, in order to assess whether an expensive simulation software tool was really needed. A deterministic model may be more user-friendly, depending on the number of replicates needed to assess the impact of each input parameter.

Input data included yearly number of the most common between-farm contacts that may potentially introduce each of the chosen diseases on each theoretical farm. These contacts included live animals, animal transport vehicles, deadstock collectors, visits by veterinarians, animal technicians performing artificial insemination (AI technicians) and hoof trimmers.

The predicted yearly numbers of different contacts were obtained from farmers’ organisations and official records.

A literature review did not provide a sufficient basis for the other necessary input parameters and, hence, expert opinion of eight of the authors was used for the selection of relevant diseases and probability estimates. The experts were selected by the first author, from the National Veterinary Institute and the Swedish University of Agricultural Sciences, the organisations providing most of the research and advice on bovine and porcine infectious diseases in Sweden, with the aim to create an expert group with a combination of solid scientific and clinical experience. The basis presented to the experts for the selection of diseases was infections that are important for cattle and pig production in the EU, and where the experts had available scientific data and personal experience on which to base their estimates. Input parameters included the probability of introduction of each disease via different between-farm contacts and the expected risk reduction by each biosecurity measure.

The biosecurity measures evaluated were those commonly recommended to farmers: quarantine for all introduced animals (3 weeks for pigs and 4 weeks for cattle, based on current practice on Swedish farms), not allowing livestock truck drivers and deadstock collectors into the animal building (biosecurity lock for loading animals and isolated outside area for animal carcasses), and providing protective clothing and boots for all visitors, plus a farm-specific hoof-trimming crush. To provide a reasonable overview of the use of the model tool, the measures were combined in two different scenarios for each herd.

### Cattle

The infectious agents used in the models were bovine respiratory syncytial virus (BRSV) and bovine coronavirus (BCoV). Three theoretical farm examples were used, to reflect the most common production systems in Sweden: one dairy farm, one specialised calf fattening farm and one suckler cow farm. The dairy farm was set to include 180 milking cows, the two beef farms had 120 fattening calves and 65 suckler cows, respectively.

### Pigs

The infectious agents used in the models were *Brachyspira hyodysenteriae* causing swine dysentery and *Mycoplasma hyopneumoniae* causing swine enzootic pneumonia. The theoretical farm examples used were one specialised fattening herd with 1600 fattening pigs and one farrow-to finish herd with 484 sows where it was assumed that all pigs were reared to market weight on the farm. No specialised piglet-producing herd was included as, from the aspects relevant to this study, it would have been fairly similar to the farrow-to-finish herd.

### Scenarios

Two different scenarios, baseline and low-risk, were used to reflect different practices of between-farm contacts, as adapting contact patterns can also be seen as part of a farm’s biosecurity strategy.

Information about typical farms and farm practices was supplied by the Swedish Animal Health Services for pig and beef herds, and by Växa Sweden for the dairy herd.

The baseline scenario included the predicted yearly contacts in a typical farm of each type. In the low-risk scenario, it was assumed that artificial insemination (AI) in the dairy herd was performed by someone on the farm (i.e. no visit from an AI technician) and that replacement heifers were bred on the farm. In the low-risk scenario for the suckler herd, the number of introduced animals was reduced. In the low-risk scenario for the pig herds and the calf fattening herd, the number of source herds for live animals was reduced. Moreover, in this scenario a lower prevalence estimate was used for the cattle diseases as it was assumed that the farmers chose to mainly buy animals during the outdoor season when the prevalences of the selected diseases are lower.

The estimated numbers of yearly between-farm contacts for each farm in the two scenarios are specified in Table 1a, b.

**Table 1:** Theoretical number of different contacts per year in 5 example herds

a. Examples included three cattle herds and two pig herds. Input values used in the baseline scenario					
Contact	Dairy herd 180 milking cows	Suckler herd 65 cows	Calf fattening herd 120 calves	Farrow-to-finish herd 484 sows	Specialised fattening herd 1600 pigs
Introduced live animals^a^	10	2	108, in 18 batches	200, in 20 batches	5200, in 26 batches
Animal transport vehicles^b^	25	3	20	60	52
Deadstock collections	17	2	3	25	5
AI technician	280	NA^c^	NA	NA	NA
Veterinarian	20	2	4	12	6
Hoof trimmer	2	1	NA	NA	NA
b. Examples included three cattle herds and two pig herds. Input values used in the low-risk scenario					
Introduced live animals^a^	1	1	100, in 5 batches	100, in 10 batches	5200, in 13 batches
Animal transport vehicles^b^	20	3	16	50	26
Deadstock collections	15	2	3	20	5
AI technician	0	NA^c^	NA	NA	NA
Veterinarian	20	2	4	12	6
Hoof trimmer	2	1	NA	NA	NA

^a^ If number of batches not specified, animals could be introduced from any herd and no sourcing from a limited number of herds was assumed

^b^ Total number of transport vehicles, including those that collect animals from the farm

^c^ NA = Not applicable

### Input parameters

The expert opinions were elicited by group discussions led by the first author and held separately for the cattle and pig experts, respectively. Within the two expert groups, each disease was discussed separately, with each route of disease introduction and the effect of respective biosecurity measures discussed in a systematic order. The outline of the model where the input parameters were to be used was explained to the experts. For probability of introduction via different routes, the experts were instructed to take different levels of compliance into account. For each parameter, maximum and minimum values were chosen first, by open discussion between all the four experts on each animal species. Subsequently a probability distribution was decided. If the experts agreed that the relevant parameter was just as likely to equal any value between maximum and minimum, a uniform distribution was chosen, and if a most likely value was put forward, a Beta pert distribution was chosen. Scientific data and the experts’ own (clinical and field research) experiences were discussed in detail in order to agree on each parameter. All input parameters were subsequently re-evaluated individually by each expert, with the explicit instruction to review the previously agreed values. All estimates (including probability distributions) of probability of introduction and percentage risk reduction are listed in Table 2, while the disease prevalences used are given in Table 3. The probability of introduction assumes that the contact comes directly from an infected farm. The varying of the figures for within-herd prevalence between one infected animal and a maximum number of concurrently infected (and infectious) animals in a uniform distribution was based on the assumption that the contact could occur in any phase of the infection. Maximum prevalence values were adjusted by the experts from published data [18–22], based on their own experience, to obtain a value representative of the maximum proportion of infected animals in the incubation or excreting phase of the infection, at the same point in time, in a Swedish herd.

**Table 2:** Probability input parameters

	BRSV	BCoV	SD	M. hyo
Contact	%	%	%	%
Animal transport vehicles^a^	0-30-80 (Beta Pert)	1-40-95 (Beta Pert)	0-3-40 (Beta Pert)	0-40 (uniform)
Deadstock collectors^a^	0-3-80 (Beta Pert)	0-5-95 (Beta Pert)	0-0.1 (uniform)	0-0.1 (uniform)
Visitor (vet, AI technician)^a^	0-40-80 (Beta Pert)	1-50-95 (Beta Pert)	0-0.1-5 (Beta Pert)	0-0.01-1 (Beta Pert)
Hoof trimmer^a^	0-40-80 (Beta Pert)	1-80-95 (Beta Pert)	NA^c^	NA
Biosecurity measure				
3–4 weeks’ quarantine^b^	0-50-99 (Beta Pert)	0-20-99 (Beta Pert)	20-50-50.1 (Beta Pert)	70-89-90 (Beta Pert)
Biosecurity lock for loading, ventilation off while truck outside	50-90 (uniform)	50-90 (uniform)	100	80-90-99 (Beta Pert)
Isolated area for carcasses	99-99.5 (uniform)	99-99.5 (uniform)	100	100
Protective clothing provided for visitors	50-80 (uniform)	50-75 (uniform)	90-99-100 (Beta Pert)	95-100 (uniform)
Farm provides hoof trimming crush	50-80 (uniform)	50-80 (uniform)	NA	NA

Introduction of bovine respiratory syncytial virus (BRSV) and bovine coronavirus (BCoV) in cattle herds, swine dysentery (SD) and *Mycoplasma hyopneumoniae* (M. hyo) in pig herds, via different contacts that come directly from an infected farm). Level of risk reduction by different biosecurity measures. Percentage figures represent minimum-most likely-maximum values or minimum-maximum values. Probability distributions shown in brackets

^a^ assuming contact with diseased animals, thus multiplied by herd prevalence in the models

^b^ 3 weeks in pig herds, 4 weeks in cattle herds

^c^ NA = not applicable

**Table 3:** Prevalence input parameters

	BRSV	BCoV	SD	M. hyo
Herd prevalence (%)	0.5-20-50 in^a^	0.5-30-50 in	0-4^b^	50-98
	0.5-2-50 out	0.5-3-50 out	0-0.5 (gilt)	40-70 (gilt)
	(Beta Pert)	(Beta pert)	(uniform)	(uniform)
Within-herd prevalence	1/n^c^-1	1/n-1	1/n-0.5	1/n-0.4
	(uniform)	(uniform)	(uniform)	(uniform)

Estimates used for the prevalence of bovine respiratory syncytial virus (BRSV) and bovine coronavirus (BCoV) in cattle herds, and swine dysentery (SD) and *Mycoplasma hyopneumoniae* (M. hyo) in pig herds. Percentage figures given represent minimum-most likely-maximum values or minimum-maximum values. Probability distributions shown in brackets

^a^ in = indoor season, out = pasture season

^b^ Different herd prevalences are given for ordinary herds and gilt-producing herds, as the latter have a lower prevalence (free from dysentery, but might be infected during transport)

^c^ n = number of animals in an average source herd for each specific animal type (gilt producers 765 animals, grower producers 3000, dairy heifer producers and bull calves 500, beef heifers 900)

### Models

The calculations were performed in a spreadsheet. A risk assessment model structure assuming independent probabilities was used [23]. The probability of introduction of a specific disease agent via a certain contact, R(contact), was calculated as:

$$ \mathrm{R}\left(\mathrm{contact}\right)=\mathrm{P}{1}^{*}\mathrm{P}2 $$

Where P1 = the probability of introduction via this contact with no mitigation, and

P2 = 1-effect of the biosecurity measure (i.e. probability of failure of a certain biosecurity measure, see Table 2 for the estimated effect of each measure).

For indirect contacts (people and vehicles) P1 was calculated as the probability of encountering the infectious agent (=herd prevalence) * probability of transmission via the specific contact (see Tables 2 and 3).

For live animals P1 was calculated as herd prevalence*within-herd prevalence.

The yearly risk of introducing each disease via each indirect contact type (R(year)) was calculated as:

$$ \mathrm{R}\left(\mathrm{year}\right)=1\hbox{-} {\left(1\hbox{-} \mathrm{R}\left(\mathrm{contact}\right)\right)}^{\times } $$

Where x = number of each type of contact during a 1-year period.

For live animals that were moved in batches, the yearly risk was calculated as (1 ‐ (1 ‐ herd prevalence)^n batches^) × (1 ‐ (1 ‐ within ‐ herd prevalence)^n animals/batch^) to account for the limited number of source herds, with subsequent multiplication by (1-the effect of quarantine measures). Here it was assumed that all animals in one batch were sourced from the same herd at the same point in time. Different batches could come from different source herds or the same herd at different times (when the infection status of the source herd might be different).

Each route of introduction was calculated separately and subsequently combined to describe the yearly risk of introducing each disease (R(total)) depending on different biosecurity measures as:

$$ \mathrm{R}\left(\mathrm{total}\right)=1\hbox{-} {\left(1\hbox{-} \mathrm{R}\left(\mathrm{i}\right)\right)}^{*}\left(1\hbox{-} \mathrm{R}\left(\mathrm{j}\right)\right) $$

Where R(i-j) = yearly risk of introduction (as calculated previously) via each contact type, with or without biosecurity measures. All equations are detailed in Table 4.

**Table 4:** Equations used for calculating the probabilities included in the model

	Yearly risk of introduction (R) via:	Input parameters	Equation
1	Individual live animals	Herd prevalence (HP), within-herd prevalence (WHP), animals introduced yearly (n)	R = 1 ‐ (1 ‐ (HP*WHP))^n^
2	Individual live animals, despite biosecurity	HP, WHP, n, effect of quarantine (Q)	R = 1 ‐ (1 ‐ (HP*WHP)*(1 ‐ Q))^n^
3	Animals in batches	HP, WHP, n, yearly number of batches (batch)	R = (1 ‐ (1 ‐ HP)^batch^) × (1 ‐ (1 ‐ WHP)^n/batch^)
4	Animals in batches, despite biosecurity	HP, WHP, n, batch, Q	R = (1 ‐ (1 ‐ HP)^batch^) × (1 ‐ (1 ‐ WHP)^n/batch^)*(1 ‐ Q)
5	Animal transport vehicles	HP, probability of transmission via transport (trp), yearly transports (n)	R = 1 ‐ (1 ‐ (HP*trp))^n^
6	Animal transport vehicles, despite biosecurity	HP, TRP, n, effect of biosecurity routine (biosec)	R = 1 ‐ (1 ‐ (HP*trp)*(1 ‐ biosec))^n^
7	Deadstock collector	HP, probability of transmission via deadstock collector (dead), yearly collections (n)	R = 1 ‐ (1 ‐ (HP*dead))^n^
8	Deadstock collector, despite biosecurity	HP, dead, n, biosec	R = 1 ‐ (1 ‐ (HP*dead)*(1 ‐ biosec))^n^
9	AI technician	HP, probability of transmission via technician (AI), yearly visits (n)	R = 1 ‐ (1 ‐ (HP*AI))^n^
10	AI technician, despite biosecurity	HP, AI, n, biosec	R = 1 ‐ (1 ‐ (HP*AI)*(1 ‐ biosec))^n^
11	Veterinarian	HP, probability of transmission via veterinarian (vet), yearly visits (n)	R = 1 ‐ (1 ‐ (HP*vet))^n^
12	Veterinarian, despite biosecurity	HP, vet, n, biosec	R = 1 ‐ (1 ‐ (HP*vet)*(1 ‐ biosec))^n^
13	Hoof trimmer	HP, probability of transmission via hoof trimmer (hoof), yearly visits (n)	R = 1 ‐ (1 ‐ (HP*hoof))^n^
14	Hoof trimmer, despite biosecurity	HP, hoof, n, biosec	R = 1 ‐ (1 ‐ (HP*hoof)*(1 ‐ biosec))^n^
15	All contacts	Equations 1 (or 3), 5, 7, 9, 11, 13	R = 1 ‐ (1 ‐ Equ1)*(1 ‐ Equ5)*(1 ‐ Equ7)*(1 ‐ Equ9)*(1 ‐ Equ11)*(1 ‐ Equ13)
14	All contacts, despite biosecurity	Equiations 2 (or 4), 6, 8, 10, 12, 14	R = 1 ‐ (1 ‐ Equ2)*(1 ‐ Equ6)*(1 ‐ Equ7)*(1 ‐ Equ10)*(1 ‐ Equ12)*(1 ‐ Equ14)

Two separate versions of the model were created for each disease in each type of herd, based on the two different scenarios. The model was created in Microsoft® Excel (Microsoft Co., Redmond USA) and Monte Carlo simulations were performed in @Risk (Palisade Co., Ithaca, USA). Each simulation was run in 10 000 iterations.

In order to assess the value of the stochastic aspect of the model, deterministic model versions were also built. In these model versions, all input parameters were fixed at the most likely value or, where a uniform distribution had been used, the average of the maximum and minimum value.

Sensitivity analysis of the effect of the various input parameters was performed using tornado graphs as well as changing the fixed input values.

## Results and discussion

The results appear to be in accordance with the underlying mechanisms that influence the risk of disease introduction to different types of farms.

### Cattle

**Fig. 1:**
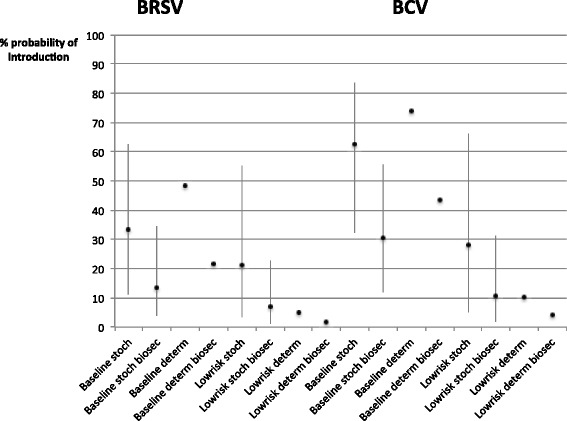
Yearly risk of disease introduction in a fictitious beef suckler herd. Model outputs for bovine respiratory syncytial virus (BRSV) and bovine coronavirus (BCV) as calculated in one stochastic (stoch) and one deterministic (determ) model based on two scenarios. (baseline and low-risk contact patterns, respectively), with and without mitigating biosecurity (biosec) measures. For stochastic models, the 5th and 95th percentiles of output is shown by the grey bar. Black dots represent median output values, whereas for deterministic models only fixed output values are shown

The results for the yearly risk of introduction of each disease in the dairy and calf fattening herds in the stochastic versions of the model are shown in Table 5. The results from the beef suckler herd are illustrated in Fig. 1. This figure also includes outputs from the deterministic model versions. Overall, the results indicate risk reducing effects of biosecurity measures as well as of changed contact patterns.

**Table 5:** Yearly risk of introduction of cattle diseases

Scenario/Model	Biosecurity measures	Dairy herd 180 milking cows	Calf fattening herd 120 calves
BRSV		%	%
Baseline	No	100 (99.96–100)	98.85 (74.86–100)
	Yes	99.99 (91.63–100)	65.70 (31.86–89.43)
Low-risk	No	75.82 (19.09–99.21)	61.00 (13.37–96.05)
	Yes	32.99 (5.83–76.20)	27.30 (4.72–66.55)
**BCoV**			
Baseline	No	100 (100–100)	99.86 (90.90–100)
	Yes	100 (99.63–100)	85.35 (54.71–97.67)
Low-risk	No	80.85 (22.91–99.54)	69.86 (17.30–98.15)
	Yes	39.12 (7.57–80.60)	38.50 (7.36–79.44)

Model outputs for bovine respiratory syncytial virus (BRSV) and bovine coronavirus (BCoV) in two example cattle herds, as calculated in a stochastic model, based on two scenarios (baseline and low-risk contact patterns, respectively), with and without application of biosecurity (quarantine for new animals, protective clothes for visitors, hygiene lock for loading/unloading, isolated deadstock collection area). Median result and 5th to 95th percentiles (in brackets) are given

The outputs in the baseline scenario for the dairy herd in particular, but also the calf fattening herd, indicate a very high risk of introduction, regardless of the application of biosecurity measures. This is due to the high maximum estimates for herd prevalence and within-herd prevalence in combination with high contact rates and a high maximum probability of introduction via these contacts. These results could be seen as realistic for a farm located in the high-prevalence areas in the southern parts of Sweden [24], with a high cattle density, but would not be realistic for farms in other areas. Even in herds with a high number of visitors (mainly AI technicians) in areas with a high prevalence, a protective effect of biosecurity measures has been noted [6], demonstrating that a high risk estimate is not absolute. The minimum values were substantially (50 %) lower than the 5^th^ percentile, reflecting a lower probability if biosecurity measures were diligently applied. The lower probability of introduction as well as the larger effect of biosecurity measures in the low-risk scenario reflects the effect of lower contact frequencies as well as a lower herd prevalence of the diseases. This effect is also seen in both scenarios for the suckler herd, that had lower frequencies for most contact types.

A high number of contacts may outweigh the effect of a reduced risk for each contact, as seen when comparing the scenarios and the different herds.

Farmers may adapt their routines based on knowledge of geographical and temporal disease prevalence. When using the model, this could be illustrated by lowering the input value for herd prevalence. Moreover, farmers can reduce risks by changing contact patterns or focusing biosecurity measures on the contacts with the highest risk for their individual farm, depending on production routines. This is reflected in the low-risk scenarios, and could be used in a discussion with the farmer by changing each individual input based on what is feasible on his/her farm and then comparing the outputs. Presenting the results in a diagram such as Fig. 1 may be most useful for a stochastic model, where the range in output can easily be demonstrated and point estimates not given too much weight, thus avoiding projection of a false image of certainty in the results. To achieve the same illustrative effect in a deterministic version of the model, the relevant input values would need to be varied in many repeats of the model, and all the different outputs shown in a diagram.

### Pigs

The results for the yearly risk of introduction of each disease in each herd type in the different model versions are shown in Table 6. For the bacterial pig diseases, estimates of disease introduction via indirect transmission routes were much lower than for the more transmissible viral bovine diseases. Biosecurity measures as well as changes in contact rates reduced the risk of introducing these diseases. However, a high number of introduced live animals in combination with a high herd prevalence made the estimated probability of introduction of *Mycoplasma hyopneumoniae* without biosecurity measures very high for both scenarios in both herds. For dysentery, with a much lower estimated herd prevalence, much lower probabilities of introduction were seen for both scenarios in both herds.

**Table 6:** Yearly risk of introduction of pig diseases

Scenario/Model	Biosecurity measures	Farrow-to-finish herd 484 sows	Specialised fattening herd 1600 pigs
SD		%	%
Baseline	No	11.43 (2.88–31.30)	45.35 (5.66–70.00)
	Yes	2.33 (0.20–4.92)	22.18 (2.69–35.75)
Low-risk	No	8.17 (1.88–25.58)	26.32 (2.98–45.35)
	Yes	1.18 % (0.10–2.52)	12.50 (1.37–22.03)
**M. hyo**			
Baseline	No	100 (98.37–100)	100 (100–100)
	Yes	53.51 (17.20–85.03)	55.86 (18.34–87.56)
Low-risk	No	99.99 (98.30–100)	100 (99.99–100)
	Yes	54.24 (17.01–86.46)	38.12 (15.09–67.61)

Model outputs for swine dysentery (SD) and *Mycoplasma hyopneumoniae* (M. hyo) in two example pig herds, as calculated in a stochastic model, based on two scenarios (baseline and low-risk contact patterns, respectively), with and without mitigating biosecurity (quarantine for new animals, protective clothes for visitors, hygiene lock for loading/unloading, isolated deadstock collection area). Median result and 5th to 95th percentiles (in brackets) are given

Most outputs from the stochastic model versions had very wide ranges and skewed distributions. To reduce the probability to the 5^th^ percentile or the minimum output value would require very strict application of all measures in order to achieve the very highest effect of all such measures. If this is deemed realistic on a particular farm, input parameters for the effect of the relevant biosecurity measures could be changed accordingly and the resulting changes in model outputs discussed with the farmer.

Adapting the pattern of live animal contacts in order to reduce certain risks is also an important aspect, where the stochastic outputs may be informative as a basis for discussion. However, a manipulation of each input based on possible changes in contact patterns and application of biosecurity measures could also be used.

It should be noted that in particular for swine dysentery, the results are only theoretically applicable to Swedish herds since the disease has been eradicated from nucleus and multiplying herds [20].

### Factors affecting the results

The differences in output for each disease and each farm type reflect the large differences in the number of contacts as well as the differences in prevalence of the specific diseases. These were the two most important factors affecting the outcome of the models, with disease prevalence being the most important. The most frequent contact type and the estimated risk for this contact was almost as important as disease prevalence when the effect of biosecurity measures was not included. When this effect was included, logically, the size of the risk reduction by the biosecurity measure affecting the risk for the most frequent contact type became important as well.

The risk of introduction also depends largely on the infectious agent. This is reflected in the different results for the different diseases that, although all contagious, have different probabilities of transmission via each contact as well as different prevalence estimates. When applying biosecurity measures, disease prevalence was the single most important factor affecting the outcome. In the presence of a regional disease control programme, the risk is reduced for all herds. However, for some herds biosecurity in the form of reduced/planned contacts and specific on-farm biosecurity measures may be needed to substantially affect the risk.

### Model inputs

Input data based on scientific studies are usually preferable, but were not available in this case. It is very difficult to use field studies for the assessment of the effect of separate biosecurity measures for separate diseases. Meanwhile, experimental studies will not provide data directly applicable to the field situation. On the other hand, expert elicitations can be quite resource demanding and there is always a risk of bias. Still, expert opinion was used in this study, making an effort to combine experience from the field with literature data to obtain realistic estimates and based on a structured discussion to avoid adjustment and anchoring [23].

The very wide output ranges are mainly due to the large range in the input values that in turn reflect the uncertainty and/or variability of these estimates. For example, variations in regional as well as individual herd immunity affect both prevalence and risk of introduction and applying the model to a specific region would allow more precise prevalence figures. Some of the uncertainty might be overcome by further studies to obtain detailed data on which to calculate the probability of introduction via various routes in a specific production system in a specific region. However, a lot of the variation is probably due to the inherent variability in biological systems as well as in human behaviour that affects the probability of introducing infectious agents via different contacts as well as the expected mitigating effect of biosecurity measures. Hence, even if uncertainty may be removed by further studies, the range in some parameter inputs, particularly the effect of biosecurity measures relying on the compliance of many people, would still be wide due to variability. This variation may be reduced, but not entirely eliminated, by efforts to improve compliance, which is a crucial aspect of successful biosecurity. When using the model on an individual farm, some of the input parameters could be changed based on the assessment of how strictly a biosecurity measure might be applied on this particular farm.

The probability of disease introduction may have been overestimated both in the input values and in the calculations, as it was assumed that a visit to an infected farm would always lead to the estimated probability of introduction (see Table 2). Moreover, each repeated sourcing from the same herd was treated as a “new” source of animals (as the herd’s disease status could change over time). This may have resulted in unrealistically high output figures. However, the exact risk figures are not very interesting per se, it is the comparison between outputs when manipulating some of the inputs that may be useful for optimising biosecurity in an individual herd. The infectious agents used as examples in this study could be replaced with others, if the individual farmer or veterinarian believe other examples would be more relevant. There is however no need to apply the model to every conceivable disease that could threaten the farm, as the purpose would be to identify the points for improvement of the farm’s general biosecurity. Thus, two examples of diseases that are present in the region but not in the individual farm would suffice.

The rates of different contacts vary between farms and thus these model inputs would need to be adjusted for the individual farm. Studies on contact rates in Swedish livestock holdings have shown large variations depending on farm type [25]. Varying the number of each specific contact did not prove meaningful for this study, as it would have resulted in an even larger number of model versions. Therefore fixed scenarios reflecting an average rate of contacts in each type of herd versus an adaptation of contacts (as would be advised for risk reduction) were used instead. The difference in results between the baseline and the low-risk scenario reflects the effect of reducing and adapting contacts in place and time (sourcing animals from fewer herds and/or during a period of lower disease prevalence).

As the purpose of the study was mainly to illustrate possible effects of the measures included in most recommendations for on-farm biosecurity, some contacts and potential biosecurity measures were left out for simplicity. However, potential biosecurity measures could be added to the model and evaluated. When adapting the model to a real farm, all contacts and other routes of transmission could be included and estimated for each disease of interest. On the other hand, including the major risks and potential risk reduction measures may be sufficient for the purpose of discussion and information.

### Usefulness of the model tool

The use of a deterministic model based on point estimates may be preferable, as it is more user-friendly and does not require any simulation software. However, as seen in Fig. 1, outputs from a deterministic model do not reflect variation and may lead to a false impression of certainty with consequently exaggerated or minimised expectations. In order to reflect the variability and uncertainty in a deterministic model, many of the input parameters would need to be varied in several scenarios that could be discussed as regards feasibility and expected costs. With a stochastic model based on sampling from a distribution of inputs, the range of outputs as well as the mean could be used as a basis for discussions about the importance of compliance and the possibility of achieving maximum effect of the different measures.

Probabilities and percentages are not easily interpreted by most people [26]. However, illustrating the results in a diagram may be more useful than simply obtaining output figures. The usefulness of the tool depends both on the farmer and the veterinary advisor and the models would need to be adapted for the specific needs of each situation.

### Compliance with biosecurity protocols

Designing good biosecurity programmes is not enough, there must be compliance in the field. There are indications that even audits and surveillance cameras are not enough to preserve good biosecurity routines in the long term [15] and that personality traits as well as education and experience are important for compliance with biosecurity protocols [27]. Financial incentives in the form of demonstrated economic benefits are perceived as important motivators by some farmers [28] whereas monetary sanctions may have the opposite effect [13]. Education and information is regarded as a key factor to the application of biosecurity routines. However, a sense of not being able to prevent disease occurrence, or that disease introduction is a random event beyond farmers’ control, can make information efforts ineffective [29].

Improving on-farm biosecurity also has implications for preventing outbreaks of exotic diseases. Most of these diseases are highly contagious but the probability of introduction still depends on many factors and may be reduced by routine biosecurity measures [30–32]. Although farmers will most likely not be very interested in preventing disease outbreaks that they believe may never happen, or perceive is the responsibility of the authorities [13, 33, 34], a perceived ability to prevent the introduction of circulating disease has been associated with a positive attitude to on-farm biosecurity measures [35]. The model presented here aims to provide farmers (and their veterinary advisors) with a sense of being able to control disease introduction to their own farm, as an incentive for improving compliance with biosecurity recommendations.

## Conclusion

The model tool proved useful for illustrating the risk of introduction of endemic diseases and the mitigating effect of biosecurity measures on farm level. It may be useful in the field if on-farm input data could be limited to farm-specific contact data and all other necessary parameters were already provided for selected disease examples. The theoretic exercise of working with the model may aid veterinary advisors in understanding farm-specific risks and in motivating farmers to improve biosecurity, as it can be tailored to each farmer’s needs and preferences.
